# Computational Study of a Motion Sensor to Simultaneously Measure Two Physical Quantities in All Three Directions for a UAV

**DOI:** 10.3390/s23115265

**Published:** 2023-06-01

**Authors:** Kamran Siddique, Yoshifumi Ogami

**Affiliations:** Department of Mechanical Engineering, College of Science and Engineering, Ritsumeikan University, 1-1-1 Noji-Higashi, Kusatsu 525-8577, Japan

**Keywords:** thermal accelerators, unmanned aerial vehicle, motion sensor, cross-axis sensitivity, microelectromechanical system

## Abstract

Cross-axis sensitivity is generally undesirable, and lower values are required for the accurate performance of a thermal accelerometer. In this study, errors in devices are utilized to simultaneously measure two physical quantities of an unmanned aerial vehicle (UAV) in the X-, Y-, and Z-directions, i.e., where three accelerations and three rotations can also be simultaneously measured using a single motion sensor. The 3D structures of thermal accelerometers were designed and simulated in a FEM simulator using commercially available FLUENT 18.2 software Obtained temperature responses were correlated with input physical quantities, and a graphical relationship was created between peak temperature values and input accelerations and rotations. Using this graphical representation, any values of acceleration from 1*g* to 4*g* and rotational speed from 200 to 1000°/s can be simultaneously measured in all three directions.

## 1. Introduction

Unmanned aerial vehicles (UAVs) offer a cost-effective and time-saving method for performing various functions, providing safety and convenience compared to traditional methods. They have a wide range of applications and are crucial to multiple industries. They can be useful in search and rescue operations [1], including disaster relief and emergency response. They can be used to deliver packages and goods to remote areas or places where road access is impossible. Additionally, UAVs can be used to inspect infrastructures such as buildings and bridges, provide high-quality images for making the right monitoring decisions [2], and monitor landslides [3].

Microelectromechanical system (MEMS)-based sensor–actuator applications continue to develop in various industries due to their improved sensitivity, accuracy, and reliability of operations, as well as their low power consumption [4]. The use of MEMS sensors for vehicular sensing is gradually increasing [5]. MEMS accelerometers have been extensively utilized in UAVs owing to their compactness, lightweight, low power consumption, and high sensitivity. Piezoelectric, capacitive, and thermal accelerometers are among the most commonly used MEMS accelerometer types in UAVs. Stephan [6] conducted a performance analysis of different accelerometer types using data sheets of 118 accelerometers from 27 different manufacturers. It was found that piezoelectric accelerometers have the highest measurement range, which can be greater than 10,000*g*. Thermal accelerometers, however, have outstanding shock limits of 50,000*g* with a low measurement range of 5*g*. Jiang [7] provides an extensive overview of piezoelectric accelerometers and mentions how these are effective for high-temperature applications, such as those encountered in aerospace, aircraft, automotive and energy industries. Thermal accelerometers, however, do not have a solid-proof mass; hence, their fabrication is simpler, and the integration of the sensor with a signal conditioning circuit is easier, resulting in improved device durability and measurement consistency [8].

**Figure 1:**
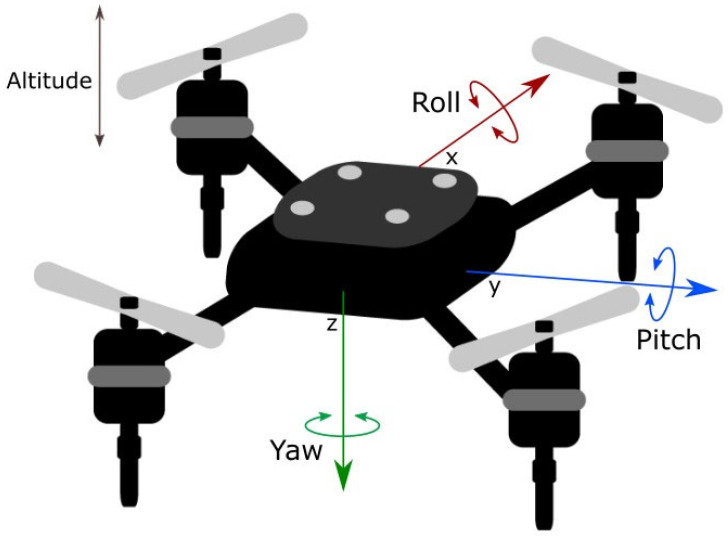
A drone’s pitch, roll, and yaw.

UAV states are generally estimated via fusing data from accelerometers, gyroscopes, and global navigation satellite systems (GNSS) to determine pitch, yaw, and roll, as shown in Figure 1. The common types of MEMS gyroscopes used in UAVs include ring lasers, fiber optics, MEMS vibrating structures, and Coriolis vibratory types. Environmental thermal fluctuations are major issues in such devices. Several researchers have presented a temperature compensation method that changes the structure of a gyroscope to decrease frequency variation under different temperatures [9,10]. This limitation of MEMS gyroscopes remains a challenge. In this study, a novel concept is proposed that involves the measurement of rotational speed in addition to acceleration, achieved by modifying a conventional thermal accelerometer that can only measure acceleration.

**Figure 2:**
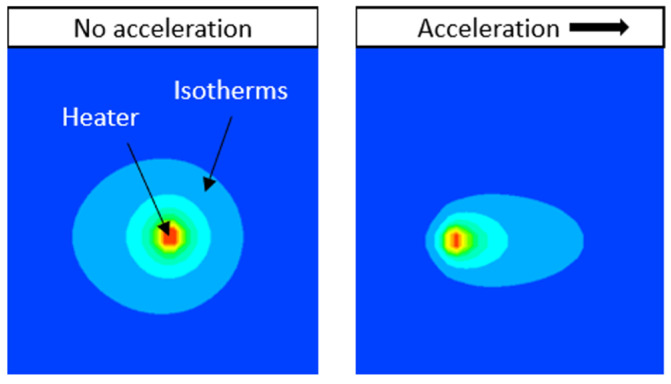
Changes in isotherms with no acceleration (**left**) and acceleration applied to the right side (**right**).

Thermal-based accelerometers offer several advantages over conventional proof-mass accelerometers. They exhibit no measurable resonance, delivering immunity to vibration; no temperature hysteresis; and excellent zero g offset stability with the added shock resistance, increasing their reliability [11]. This enhances the sensing range of the device while avoiding failure or vibrational limitations. The working principle is that a heating source creates a thermal profile inside a cavity, and a set of temperature sensors placed equidistant from the heater measures the temperature. When acceleration occurs, the sensors’ temperature values change, corresponding to the applied acceleration. This phenomenon is illustrated in Figure 2. As shown in the figure, sets of isotherms were created around the heating source, and isotherms near the heater had higher temperatures. A consistent temperature profile was obtained without applying any motion, as shown in Figure 2(left). However, when acceleration was applied in the right direction, the sets of isotherms shifted to the right (Figure 2(right)). This temperature change was detected by sensors placed on both sides of the heater and correlated with applied acceleration.

Different numerical and experimental studies have been conducted to optimize multiple-axis thermal accelerometers. Novel triple-axis thermal accelerometers were introduced by [12] and improved by [13]. However, their sensitivities were still insufficient and required further improvement. Mukherjee [14] modified the cavity structure of the device to achieve better sensitivity. Jiang [15] showed that increasing heater power enhanced the sensitivities along the X-, Y-, and Z-axes. Wang [16] achieved improved sensitivity and a wider ambient temperature measurement range in a recent study. This design can precisely detect a range of temperatures in various environments. In this study, we aimed to select the parameters with high sensitivity and resolution, as reported in previous studies.

For a dual-axis thermal accelerometer, two sets of temperature sensors are required around the two axes. In contrast, three sets of temperature sensors are required to detect temperatures around the X-, Y-, and Z-axes for a triple-axis thermal accelerometer. To reduce installation and maintenance costs, Ogami’s concept [17], which uses cross-axis sensitivity (CAS), can be used. Due to CAS, when motion is applied to a single axis, a correlating temperature change is observed on the other two perpendicular axes. According to Farahani [18], CAS is a good measure of sensor performance in response to external factors. Therefore, for high-accuracy applications, a lower CAS is expected. However, a computational study presented by Siddique [19] showed that multiple physical quantities could be measured using CAS. In this study, using X- and Y-sensor outputs, acceleration in the X-direction and rotational speed on the Z-axis were measured.

With the development of technology, multiple sensors have been incorporated into engineering devices. In micro- and insect-scaled UAVs, multiple sensors from accelerometers to magnetic sensors have been installed [20]. The installation and maintenance of a considerable number of sensors can be expensive and time-consuming. Therefore, this study introduces a method for simultaneously measuring three accelerations and three rotations in UAVs using a single-motion sensor. The measurement of rotational speed, including pitch, roll, and yaw, is crucial for controlling the flight of the device. Using fluid flow and thermodynamic principles and leveraging CAS, we derived three inverse functions from a set of axial accelerations and the rotational speeds perpendicular to them. The inverse functions could then be implemented in the computing unit of a real motion sensor.

## 2. Materials and Methods

In this study, ANSYS FLUENT 18.2 was employed to perform computational analyses. The temperature response was obtained via the simultaneous application of acceleration and rotation around the axis perpendicular to the other axis, on which the direction of acceleration was applied to the computational model. The design of a triple-axis thermal accelerometer was considered, and heating sources were incorporated into the model using C-programmed user-defined functions (UDFs). Additionally, the positions of the temperature sensors were determined using UDFs by tracking their cell IDs, which are unique to every mesh element.

The thermal accelerometer is based on heat transfer and especially on free convection heat transfer in a closed chamber. This phenomenon is governed by a Navier–Stokes equation based on the principle of conserving mass, momentum, and energy, as follows:

$$\frac{\partial \rho }{\partial t}+\nabla \left(\rho u\right)=0$$

$$\rho \left[\frac{\partial u}{\partial t}+(u\cdot \nabla )u\right]=-\nabla p+\nabla I+f$$

$$\rho C_{p}\left(\frac{\partial T}{\partial t}+u\cdot \nabla T\right)=k\nabla^{2}T$$

where ***u*** is the flow velocity vector field, ∇ is the spatial divergence operator, *p* is the pressure, *I* is the total stress tensor, and *f* is the body forces acting on the fluid. The parameters *C_p_*, ρ, and *k* are the specific heat, density, and thermal conductivity of the fluid in the cavity, respectively.

In FLUENT, a pressure-based transient solver was used along with an energy model because the flow characteristics were not highly compressible. The DEFINE_CG_MOTION UDF was applied to define linear and rotational motions. Carbon dioxide (CO_2_) was selected as a gas medium because of its high density and low kinematic viscosity. The low viscosity of CO_2_ enables a more efficient flow and results in greater sensitivity than gases with higher viscosities because high viscosity impedes gas flow [21].

**Figure 3:**
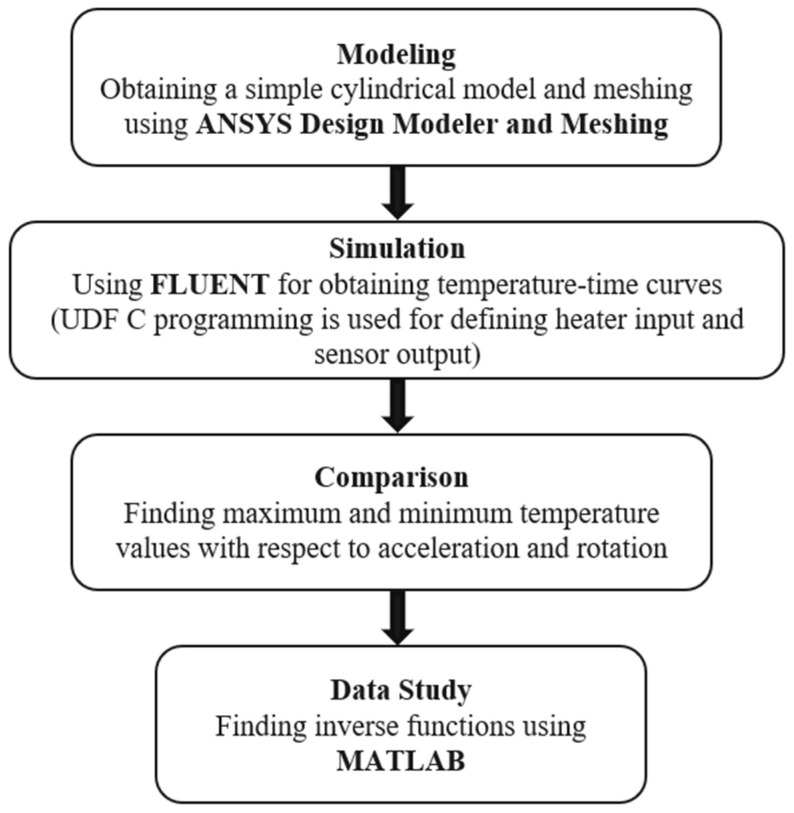
Methodology flow.

The steps involved in measuring multiple physical quantities using a motion sensor are shown in Figure 3. The first step was to generate a cylindrical model of the motion sensor with a diameter of 2 cm and depth of 1.3 cm, in which heaters and sensors were placed. Next, a mesh that served as an input to ANSYS FLUENT was developed. The next step was to input different values of acceleration and rotation and the temperature–time curves were generated. Analysis of these curves revealed the maximum and minimum temperature values, which were then linked to input acceleration and rotation values. Finally, a three-dimensional graph was drawn to visually represent the relationship between acceleration, rotation, and extreme temperature values (T_max_ and T_min_).

In thermal accelerometers, a heating source that generates temperature contours must be defined. The temperature response is generally greater when the heating power is high and the ambient temperature is low. Therefore, the differences in peak values increase. However, this results in higher temperatures inside the cavity, which can lead to the heating of the motion sensor walls. In our previous study [19], a heating power of 70 mW was considered for applications in small-scale UAVs and robots. In this study, we reduced the heating power from 70 to 40 mW, so that it can be applied to significantly smaller UAVs.

For the simulations, a time step size of 0.0042 s was considered with regard to increment velocities. The acceleration and rotation ranges were set to 1–4*g* and 250–1000°/s, respectively. For the case of acceleration, the maximum velocity for a flow time of 3 s was 117.72 m/s with an increment velocity of 0.164808 m/s at 4*g*. In contrast, for rotational velocities, a maximum linear speed of 0.174533 m/s was practically the same as the maximum increment velocity at 4*g*. The values are listed in Table 1 for comparison.

**Figure 4:**
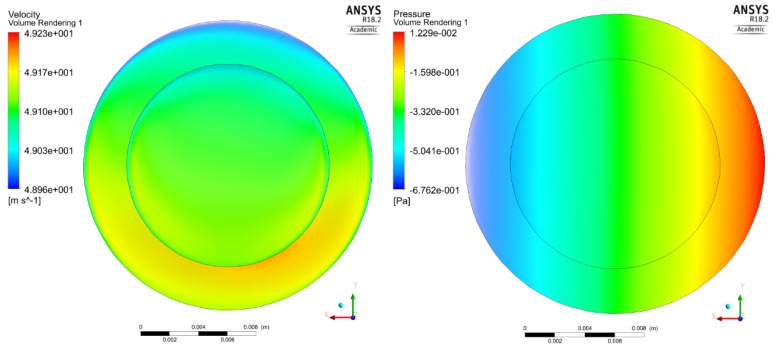
Velocity (**left**) and pressure distribution (**right**) at t = 2.5 s for the mesh with 166,675 elements.

**Figure 5:**
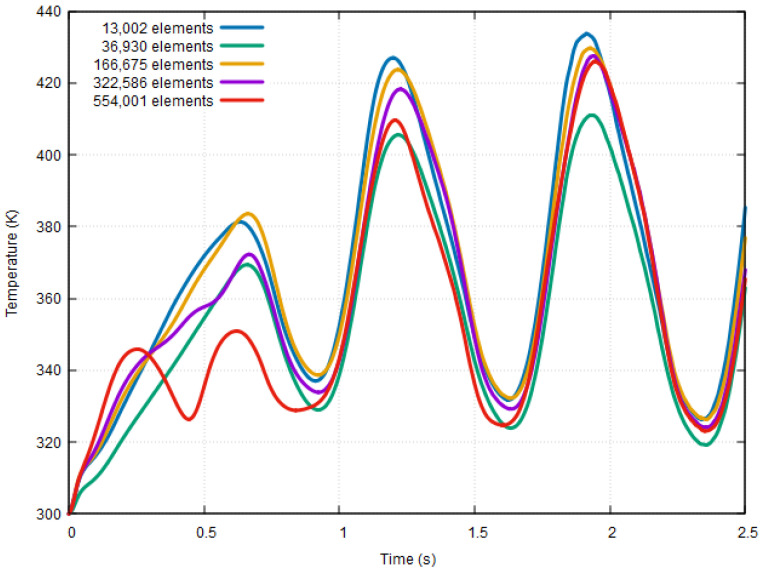
Temperature response at 500°/s around the Z-axis and 2*g* applied in the X-direction for different mesh sizes.

To validate the reliability and independence of computational simulation results, a grid-independence test was conducted because no experimental or theoretical models were available for comparison. The velocity, pressure (at sensor X11), and temperature variables were evaluated at 500°/s around the Z-axis, and 2*g* was applied to the device in the X-direction using five different meshes with varying numbers of elements. The velocity and pressure distributions (viewed from the top plane of the motion sensor) of a mesh with 166,675 elements are shown in Figure 4. It can be observed that the velocity of CO_2_ molecules was practically the same throughout the motion sensor cavity with a maximum velocity of 49.23 m/s^2^ at the instant of t = 2.5 s. Furthermore, the temperature change with respect to time for all five meshes is shown in Figure 5. The maximum values in this graph were extracted and used for comparison.

**Figure 6:**
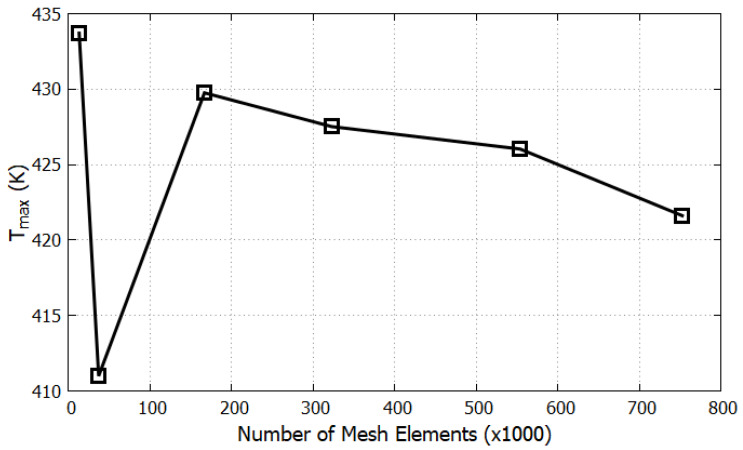
T_max_ vs. number of mesh elements.

The results of the grid-independence test, which compare the T_max_ values with respect to the number of mesh elements, are presented in Table 2 and illustrated in Figure 6.

**Figure 7:**
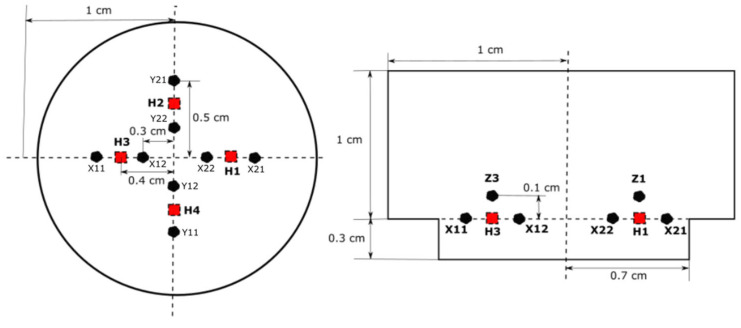
Position of heaters and sensors; cross-sectional (**left**) and side views (**right**).

**Figure 8:**
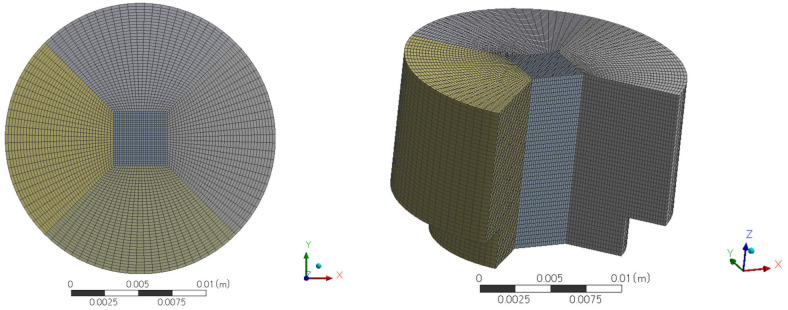
Computational mesh; top view (**left**) and isometric view of the inside of the mesh (**right**).

As shown in Figure 6, a significant increase of 4.6% in the T_max_ value can be observed between the second and third meshes. However, from the third mesh onwards, the difference in T_max_ values between the meshes was approximately 1%, which was minimal compared to the differences between the first three meshes. Therefore, a mesh with 166,675 elements was selected for future calculations because it provides a reliable solution with minimal computational requirements such as CPU time. The placement of the heaters and sensors is shown in Figure 7, and the meshing structure used in the computational study is shown in Figure 8. Four heaters (H1–H4) were placed on all four axes, 40 mm from the center of the cavity. Heaters H1 and H3 on the X-axis are surrounded by pairs of X-sensors: X21 and X22, and X11 and X12, respectively. Similarly, H2 and H4 on the Y-axis are bounded by Y-sensor pairs: Y21 and Y22, and Y11 and Y12, respectively. On the Z-axis, each heater constitutes a temperature sensor located 10 mm away from the heater in the Z-direction. The notation of the Z-sensors is such that the number denotes the heater number (e.g., Z1 around H1).

To define the relationship between two input physical quantities (PQ) and two output variables, the following equation was established:

$$\left(Output\;1,\;Output\;2\right)=ƒ\left(PQ\;1,PQ\;2\right)$$

Once this relationship was established, the next step was to find the inverse function of this relationship, which is represented as:

$$\left(PQ\;1,PQ\;2\right)={ƒ}^{-1}\left(Output\;1,Output\;2\right)$$

**Figure 9:**
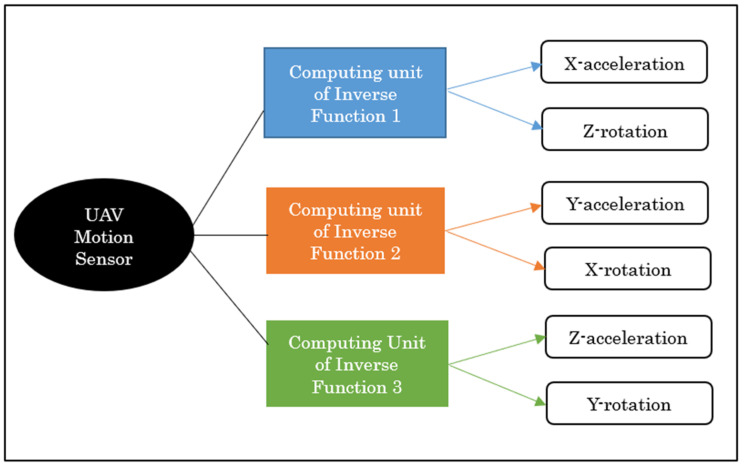
Schematic of obtaining three inverse functions for the measurement of acceleration and rotation in all three directions.

Using the above equation, any two PQs can be measured simultaneously. In this study, this relationship is calculated for two quantities in three directions. Therefore, three inverse functions must be obtained, and the data can be installed in the computing unit of the motion sensor. This is illustrated in Figure 9.

## 3. Results and Discussions

A numerical study was conducted using computational simulations to measure two physical quantities (acceleration and rotation) in all three directions. As described in Section 2, three inverse functions were obtained via computational fluid dynamics (CFD) simulations using the ANSYS Fluent 18.2 software, and each inverse function measured one acceleration (*a*) and one rotation (*ω*) perpendicular to a particular acceleration value. The inverse functions can be described as follows:

$$\left(a_{x},\omega_{z}\right)={ƒ}^{-1}\left(T_{x\max},T_{x\min}\right)$$

$$\left(a_{y},\omega_{x}\right)={ƒ}^{-1}\left(T_{y\max},T_{y\min}\right)$$

$$\left(a_{z},\omega_{y}\right)={ƒ}^{-1}\left(T_{z\max},T_{z\min}\right)$$

Cross-axis sensitivity (CAS) is a significant issue in thermal devices, including those examined in this study. To measure acceleration and rotation simultaneously, we can evaluate the peak temperature values at the axis where acceleration is applied. A previous study [12] demonstrated that temperature data from both the X- and Y-axes can be simultaneously utilized to measure the X-acceleration and Z-rotation. Therefore, we can extract data from the axis where only acceleration is applied by accounting for the CAS influence.

**Figure 10:**
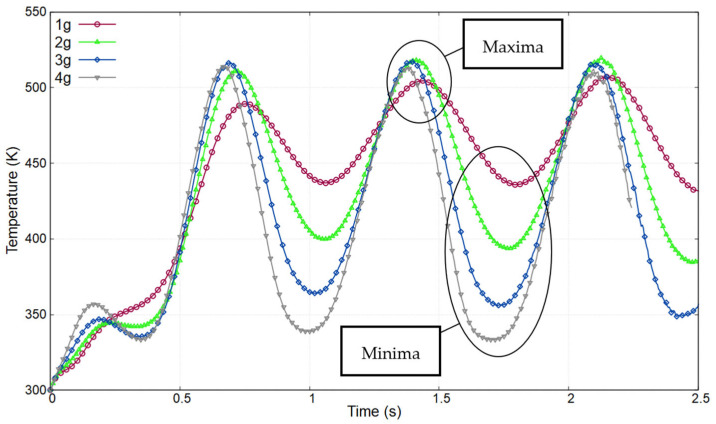
Temperature–time curve for Y12 sensor at 500°/s with varying accelerations from 1 to 4*g*.

As indicated in Figure 3 of Section 2, temperature–time curves were generated via simulations using FLUENT software. Acceleration and rotation were simultaneously applied to the cylindrical model, and the temperature graphs were analyzed to extract maximum and minimum values. To illustrate this process, Figure 10 shows the data acquired by the Y12 sensor at a rotational velocity of 500°/s and 1–4*g* accelerations. The identified peak values are listed with respect to physical input quantities (Table 3, Table 4 and Table 5).

This research study explores accelerations ranging from 1 to 4*g* (9.81–39.24 m/s^2^) and rotations ranging from 200 to 1000°/s in all three directions. As described in Section 2 and Figure 7, four heaters and four pairs of temperature sensors were positioned in all three directions. Owing to the symmetry of the structure, identical results were obtained for X11 and X21, and X12 and X22. Similarly, Y11 and Y21, and Z1 and Z3 had similarly extreme temperature values. Therefore, to obtain these extreme values, the maxima and minima of X11, Y12, and Z4 were considered. These values were then recorded for all accelerations and rotations in all three directions and correlated with the applied physical quantities of acceleration and rotation.

Because we obtained results from the computational simulation for only four data points of acceleration and rotation, we had to employ the interpolation technique to obtain more data between the upper and lower limits of input physical quantities. This was performed in MATLAB using a cubic interpolation technique, which required four data points to compute a polynomial. In this method, no constraints were present in the derivatives, unlike other interpolation techniques such as spline interpolation.

**Figure 11:**
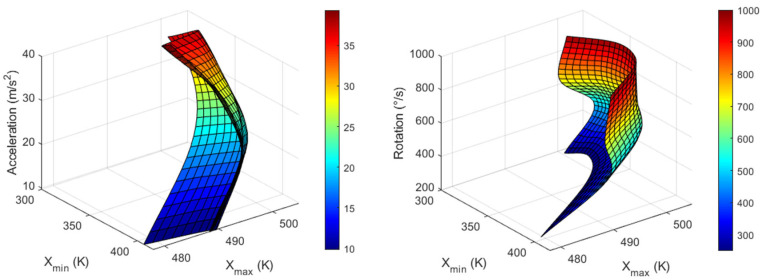
Graphs for X-acceleration (**left**) and Z-rotation (**right**) values from X_min_ and X_max_ measured around heater 1.

**Figure 12:**
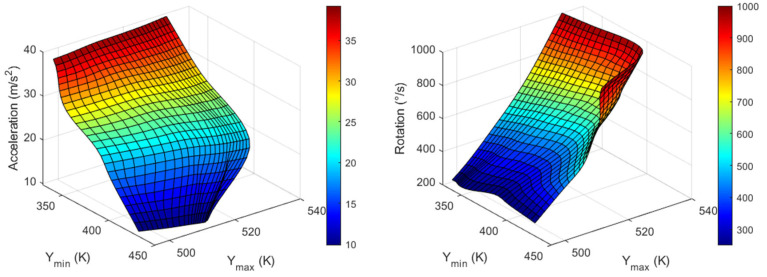
Graphs for Y-acceleration (**left**) and X-rotation (**right**) values from Y_min_ and Y_max_ measured around heater 2.

**Figure 13:**
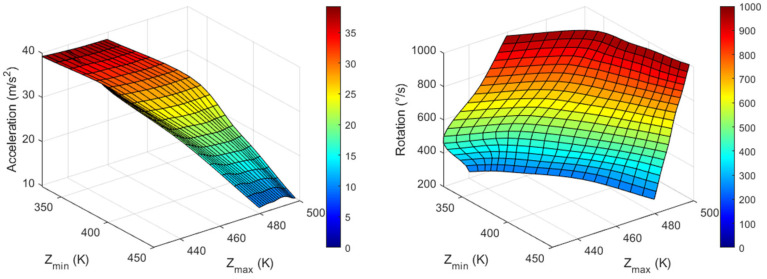
Graphs for Z-acceleration (**left**) and X-rotation (**right**) values from Z_min_ and Z_max_ measured around heater 4.

The T_max_ and T_min_ values for all three axes are listed in Table 3, Table 4 and Table 5, and the inverse functions used to obtain acceleration and rotational speed in all three directions corresponding to the measured maximum and minimum temperature values are shown in Figure 11, Figure 12 and Figure 13. Node values indicate each data point. These inverse functions were installed in the computing unit of a real thermal motion sensor.

Using the data from Figure 11, Figure 12 and Figure 13, a real thermal motion sensor can simultaneously measure all three accelerations at any value between 1 and 4*g* and rotation speeds between 200 and 1000°/s. The range of acceleration and rotational speeds can be further increased by conducting additional simulations. Furthermore, to obtain more accurate results, more data should be extracted using simulations rather than relying on interpolation techniques.

**Figure 14:**
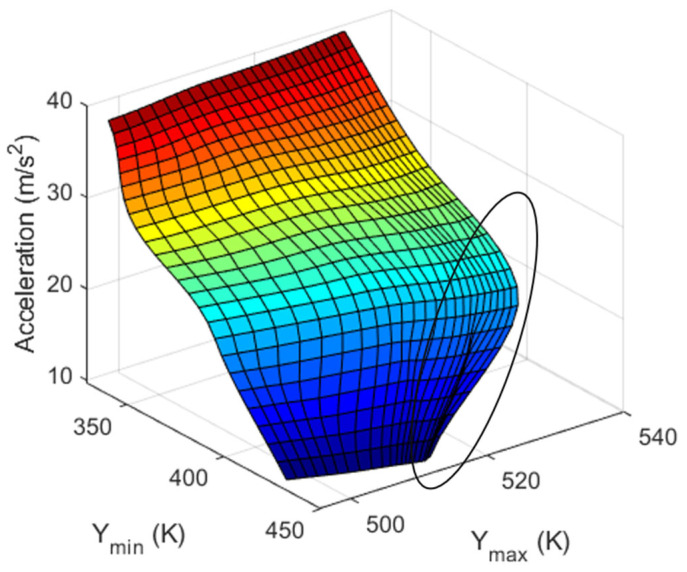
Region of multiple solutions indicated on the graph to obtain Y-acceleration using Y_max_ and Y_min_.

The main issue encountered in the aforementioned inverse functions is in the region of multiple solutions. In this region, two identical combinations of maximum and minimum temperature values generated different input physical quantities. This yielded inaccurate results. This region can be viewed by drawing a vertical line from the XY-plane parallel to the input physical quantity axis. The Y-acceleration was measured using Y_max_ and Y_min_, and this region is represented by an ellipse in Figure 14. Therefore, the results should be verified, and the parameters that generate unique solutions should be determined. This problem can be reduced by either altering the cavity shape of the sensor or changing the positions of heaters and sensors.

As mentioned in Section 2, no experimental or theoretical model is presented in this study to support or validate numerical simulations. Our future research will involve the manufacturing of a real thermal device based on the results presented in this study. However, the technique presented in this study may provide a cost-effective and time-saving method in the field of UAV sensor technology.

This method has the disadvantage of requiring both acceleration and rotation. However, considering both rotary-wing and fixed-wing UAVs, the pitch motion along the transverse or lateral axis is effective throughout the flight of the device. Furthermore, study in which more than two quantities can be measured using a single inverse function must be conducted. The idea for this is proposed below and will be explored in future research.

**Figure 15:**
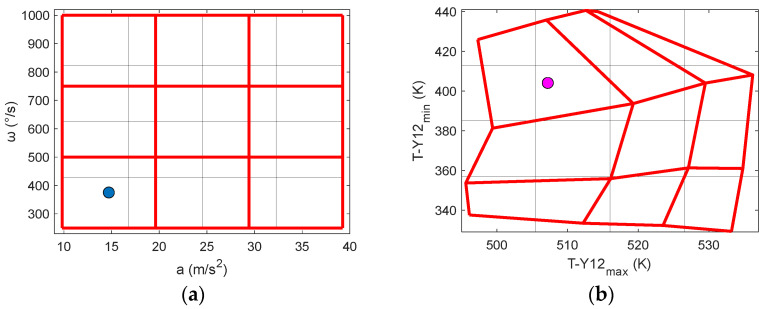
A 2D mapping of input data (**a**) and output data (**b**).

In future studies, a mapping method (2D or 3D) could be utilized to measure multiple physical quantities without using an inverse function. In our study, taking the examples of Y-acceleration and X-rotation for the 2D method, input data, i.e., acceleration and rotational speeds, were first plotted in a graph, as shown in the mapping of input data in Figure 15a (red lines). Similarly, Figure 15b (red lines) shows the output data (T_max_ and T_min_) extracted from Table 4. For real measurements, if the output (measured) values of the sensor can be obtained (plotted in Figure 15b (purple circle)), the input values for the output values can be approximately and geometrically calculated without using an inverse function, as shown in the blue circle in Figure 15a. If quadrilateral shapes overlap with each other for the output data, it will indicate multiple solutions. This technique can also be utilized in 3D to simultaneously measure three physical quantities.

## 4. Conclusions

In this study, we propose a concept using a computational simulation in which acceleration and rotational speeds of unmanned aerial vehicles (UAVs) can be simultaneously measured in all three directions using a single device. Cross-axis sensitivity, which is an error in accelerometers and gyroscopes, was utilized to achieve this goal. For X-acceleration and Z-rotation, maximum and minimum temperature values from X-sensor data were extracted and from these output values, and input quantities were measured. In a similar manner, Y-axis output data were considered for Y-acceleration and X-rotation, and Z-sensor data were considered for Z-acceleration and Y-rotation. Six graphical plots are presented for each quantity, by which any acceleration from 1 to 4*g* and rotational speed between 200 and 1000°/s can be measured in the X-, Y-, and Z-directions. The inverse function plots can then be installed in the computing unit of a real thermal motion sensor to measure the quantities.

We also proposed a new technique of 2D or 3D mapping, which we can use to effectively measure multiple physical quantities numerically from the plots of output values corresponding to their input values. This may be a better approach than using inverse function and will be a focus of our future research.

Validation using theoretical and experimental models is absent in this study and will also be a focus of our future research. A microelectromechanical system (MEMS)-based motion sensor, as described in this study, will be manufactured, and using the same flow conditions and parameters, simulation results will be validated in the future. Overall, this methodology provides a robust framework for measuring multiple physical quantities and enables researchers to gain deeper insights into their experimental data. This study provides an excellent solution for challenges faced by sensor technology in UAVs.

## Figures and Tables

**Table 1:** Maximum and increment velocities at different accelerations and rotations.

		Maximum Velocity at 3 s (m/s)	Increment Velocity (m/s)
Acceleration	1*g*	29.43	0.0412
	2*g*	58.86	0.0824
	3*g*	88.29	0.1236
	4*g*	117.72	0.1648
Rotation (°/s)	250	0.0436	0.0436
	500	0.0873	0.0873
	750	0.1309	0.1309
	1000	0.1745	0.1745

**Table 2:** Changes in variables with different numbers of mesh elements.

S	No. of Mesh Elements	V_max_ (m/s)	P_X11_min_ (Pa)	P_X11_max_ (Pa)	T_max_ (K)
1	13,002	49.23	−0.497392	−0.168213	433.7
2	36,930	49.23	−0.509235	−0.167053	411.0
3	166,675	49.235	−0.508066	−0.181941	429.8
4	322,586	49.235	−0.513125	−0.183977	427.5
5	554,001	49.24	−0.518651	−0.185735	426.0
6	752,760	49.16	−0.523673	−0.186014	421.6

**Table 3:** Data for T_X11_max_ and T_X11_min_.

**T_X11_max_**		***ω***	**250**	**500**	**750**	**1000**
	**a**					
	1*g*		477.9	488.9	488.3	489.7
	2*g*		495.0	502.6	501.1	500.7
	3*g*		500.9	504.8	501.4	501.4
	4*g*		498.4	503.3	495.5	496.7
**T_X11_min_**		***ω***	**250**	**500**	**750**	**1000**
	**a**					
	1*g*		407.1	415.7	417.3	415.7
	2*g*		359.1	373.2	379.5	380.1
	3*g*		328.3	338.5	346.1	348.9
	4*g*		312.7	316.4	320.3	322.1

**Table 4:** Data for T_Y12_max_ and T_Y12_min_.

**T_Y12_max_**		***ω***	**250**	**500**	**750**	**1000**
	**a**					
	1*g*		497.3	507.0	512.6	514.3
	2*g*		499.4	519.3	529.5	536.2
	3*g*		495.6	516.1	527.1	534.8
	4*g*		496.1	512.2	523.5	533.2
**T_Y12_min_**		***ω***	**250**	**500**	**750**	**1000**
	**a**					
	1*g*		425.9	435.8	440.6	440.0
	2*g*		381.3	393.7	404.0	408.1
	3*g*		353.7	355.9	361.3	361.0
	4*g*		337.6	333.4	332.3	329.3

**Table 5:** Data for T_YZ4_max_ and T_Z4_min_.

**T_Z4_max_**		***ω***	**250**	**500**	**750**	**1000**
	**a**					
	1*g*		483.1	490.3	497.3	500.2
	2*g*		471.4	475.5	486.3	494.3
	3*g*		452.8	454.8	471.9	486.9
	4*g*		438.9	426.8	444.1	457.3
**T_Z4_min_**		***ω***	**250**	**500**	**750**	**1000**
	**a**					
	1*g*		442.4	440.6	438.4	444.8
	2*g*		388.2	398.6	401.7	399.7
	3*g*		350.7	354.8	360.0	360.2
	4*g*		332.2	329.4	333.2	334.0
